# Factors of well-being of youth with complex medical conditions from the experience of hospitalization and convalescence: A pilot study

**DOI:** 10.1371/journal.pone.0285213

**Published:** 2023-05-04

**Authors:** Sarah Muñoz-Violant, Verónica Violant-Holz, Manuel J. Rodríguez

**Affiliations:** 1 Hospital Pedagogy in Neonatology and Pediatrics-Research Group, Universitat de Barcelona, Barcelona, Spain; 2 Department of Didactics and Educational Organization, Faculty of Education, Universitat de Barcelona, Barcelona, Spain; 3 International Observatory in Hospital Pedagogy, Universitat de Barcelona, Barcelona, Spain; 4 Department Biomedical Sciences, Institute of Neurosciences, School of Medicine and Health Sciences, Universitat de Barcelona, Barcelona, Spain; 5 August Pi i Sunyer Biomedical Research Institute (IDIBAPS), Barcelona, Spain; Al-Ahliyya Amman University, JORDAN

## Abstract

Well-being in children with a complex medical condition (CMC) impacts the way they view and communicate with their immediate environment as well as their development, and it is thus necessary to inquire about the contextual issues and different needs that a CMC carries. This pilot study aimed to identify factors of pediatric well-being from the experience of hospitalization and convalescence of youth with CMC and their caregivers, in a cross-sectional analysis using a selective methodology complemented by an indirect observational methodology. We analyzed the quality of life and well-being of youth with CMC using a validated KINDL^R^ questionnaire. We collected 35 surveys: 11 from youth with CMC and 24 from caregivers from Spain. We focused the analysis on sociodemographics, well-being perceptions, and coping strategies variables. The results show that children aged between 3 and 6 years and their caregivers scored physical well-being the lowest out of all dimensions of well-being, and they scored family well-being the highest. Moreover, youth between the ages of 7 and 17 years and their caregivers scored school-related well-being the lowest. Coping strategies to deal with stressful situations differ between children and caregivers. While children mainly engage in social withdrawal, caregivers engage in cognitive restructuring and expressing emotions. However, we did not find a relationship between coping strategies and well-being perceptions. These results highlight the need to facilitate communication spaces with both families and health professionals where the voice of children is considered.

## Introduction

The number of children with complex medical conditions (CMC) continues to increase due to the reduction in infant mortality, access to new technologies, and the improvement in social and health markers [1, 2]. Following Cohen et al.’s [3] definition of medical complexity, CMC are here understood as the medical fragility and intensive care needs of children presenting either a multisystemic disease (congenital or acquired) or a severe neurological condition, both leading to a functional impairment of daily living. This group of patients also includes the pediatric population with special health care needs due to increased risk of chronic physical, developmental, behavioral, or emotional conditions [4]. Chronic conditions are characterized by four domains [3], namely needs, chronic conditions, functional limitations, and healthcare use, all of which contribute to health-related quality of life (HRQoL). Thirty years ago, children with these conditions could not survive; nevertheless, they can now be treated with fragile drug profiles and special care needs [3]. A 2020 study found that in Spain, around 15% of the sampled children accessing primary care had a chronic illness [5], and another previous study found that the most frequent pediatric chronic conditions were neurological (76.95%), gastrointestinal (63.78%), and respiratory diseases (61.72%), respectively [6]. In the specific case of cancer, 80% of affected European boys and girls survive until adulthood, but many of them require permanent medical follow-ups and substantial care needs [7–9], including hospitalization in intensive care units and greater technological assistance and highly specialized services [10–12].

HRQoL is a variable used to measure the impact of illness on quality of life (QoL). Previous research has shown that HRQoL is significantly low in children with CMC and their caregivers [13] due to various factors such as caregiver stress and burnout [14, 15], the hospitalization process [16, 17], and poor physical and mental health [18] caused by a condition of illness.

The hospitalization process often involves a traumatic loss of children’s daily life and significant physical discomfort and pain [19]. Consequently, these children regularly show poor well-being, psychological distress, and psychiatric symptomatology such as anxiety and depression [20–23]. Generally, the presence of secondary mental health problems leads to a higher number of hospital visits, worse treatment outcomes, and poor pain management [24]. Additionally, studies have suggested that both psychological distress and psychiatric conditions are associated with the dysregulation of the immune function in youth [25, 26].

Poor physical and mental health well-being negatively impacts the family environment, which can slow the recovery of children and adolescents with CMC [27–29]. Simultaneously, research has shown that the caregivers and family members of youth with CMC regularly experience psychological distress, post-traumatic stress, and symptoms of anxiety and depression following their children’s hospitalization [30–33]. These psychological effects among caregivers seem to be present across all diagnoses—cancer [34–36], heart diseases [37–40], and other illnesses—that require hospitalization at either intensive neonatal care units or intensive pediatric care units [41–43].

It is necessary to establish a supportive environment that considers the psychosocial needs of patients and their caregivers during the hospitalization process holistically [44]. In Spain, this environment must target improved communication with health professionals and the family members’ feelings of isolation and maladaptive emotions, such as fear and pain. Additionally, it is necessary to expand opportunities for patients for relationships with their peer group [45–49].

Another environmental factor that determines the QoL of youth with CMC is the school context, which is an opportunity for children to develop socially, emotionally, physically, and educationally [50]. Nevertheless, the school experience of children and adolescents with CMC is significantly impacted by the primary and secondary aspects of their illness, including hospitalization stays, physical and psychological discomfort, and feeling different from their peers [51, 52]. Furthermore, parental perceptions of QoL related to their children’s health can also influence school attendance [53]. An American National Survey with more than 400 children with congenital heart disease reported that they are three times more likely to miss more than 10 days of daycare/school a year [54]; consequently, school reintegration during and after treatment is paramount [55] for improving the QoL—and probably the self-esteem—of these patients. Self-esteem levels in youth with CMC are actually significantly lower than their healthy peers, particularly once hospitalization and treatment have ended [56, 57]. As low self-esteem can develop into poor satisfaction and anxiety and depressive symptomatology, it is essential to target it [58–60]. Finally, one study found self-esteem to predict the willingness to receive treatment [61].

For all these reasons, appropriate coping strategies for children with CMC and their caregivers that can ease their physical and mental health burden and improve their QoL must be investigated further. Consequently, this pilot study aimed to identify the factors of pediatric well-being from the experience of hospitalization and convalescence of youth with CMC and their caregivers. For the purpose of this study, “youth” indicates children and adolescents.

### Research questions

How does the perception of children and adolescents with CMC about the hospitalization and convalescence experience influence their well-being? How do children and adolescents with CMC perceive the experience of hospitalization and convalescence compared to their caregivers?

## Materials and methods

### Study design

**Fig 1:**
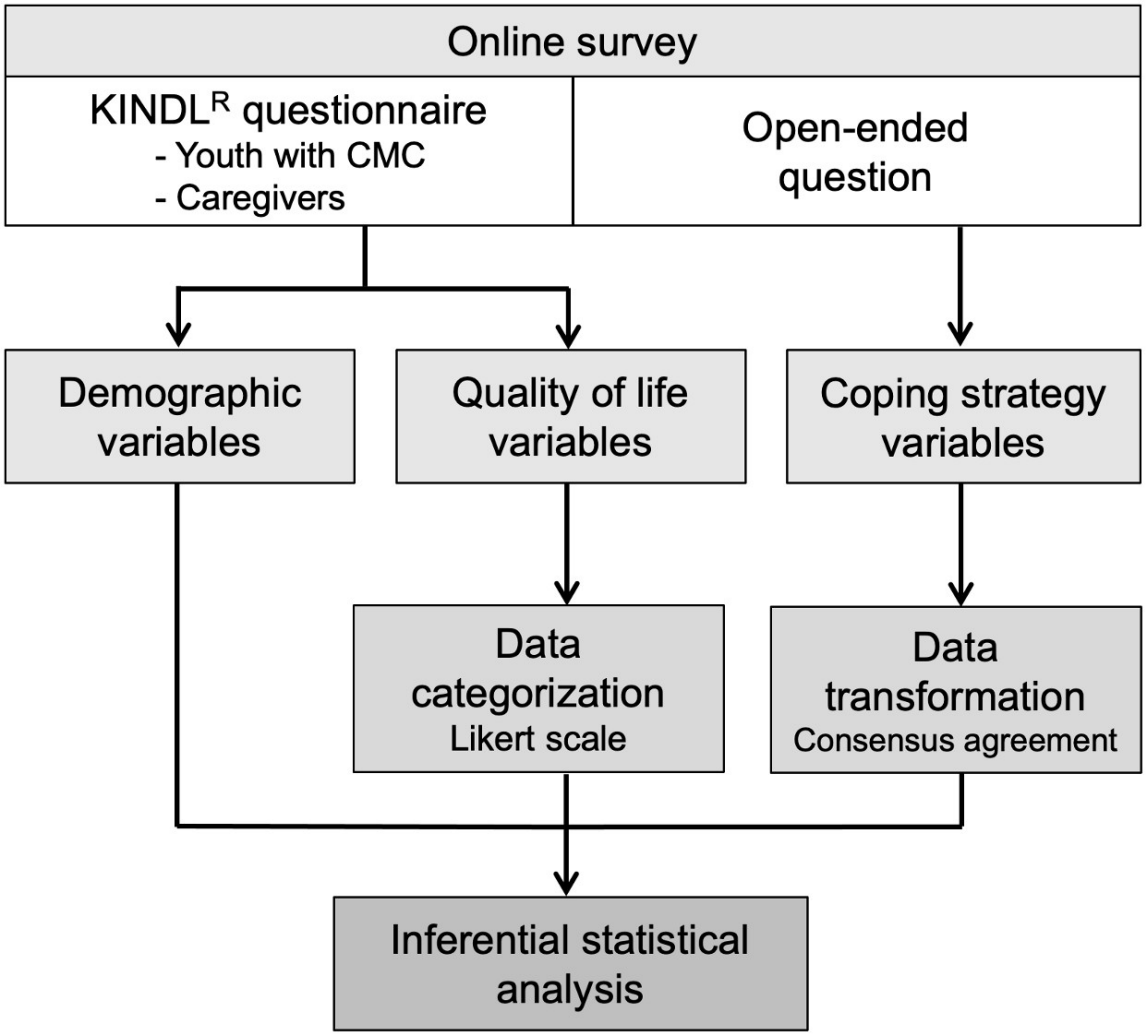
Flow chart of the research methodology of this study.

This was a cross‐sectional pilot study with a selective methodology. It used the validated KINDL^R^ questionnaire as elicitation of the responses, complemented along with an indirect observational methodology [62], and it focused on responses to open-ended questions. These responses were coded by consensus agreement [63] by the team’s three members following a well-established validated model [64]. Within the mixed-methods framework, qualitative elements (indirect observational data) were integrated with quantitative elements—the quantitative analysis techniques used, which are highly robust and suitable for qualitative data [65–67]. Fig 1 summarizes the methodological approach of the study.

### Participants

We recruited 38 participants to answer a survey through convenience sampling and screened their eligibility according to the inclusion criteria detailed in Table 1.

**Table 1:** Inclusion criteria of the study.

Inclusion criteria
Population
Participants are caregivers of youth aged 3–17 with CMC, hospitalized or not
Participants are children or teenagers aged 3–17 with CMC, hospitalized or not
Residency
Residing in Catalonia, Spain
Language
Appropriate understanding of Spanish or Catalan
Data provided
Survey fully completed

### Instruments and measurements

The survey collected sociodemographic variables and health variables as described below. We conducted a literature search to guarantee relevance to this population.

**Sociodemographic variables** for caregivers and children with CMC included the age of children with CMC, the number of children and family members at home, the presence of pets at home, children’s hospitalization state, and the maintenance of personal hobbies, among others (Table 1).

**Health variables** included the participants’ perception of QoL, well-being and disease, and coping strategies displayed to deal with stressful situations. We selected the KINDL^R^ questionnaire to measure HRQoL in children and adolescents [68]; this instrument measures children’s HRQoL through self-reporting and can be used for both healthy children and in clinical practice. We selected this questionnaire after a literature search with the following inclusion criteria: (1) self-reported; (2) availability of versions for children, teenagers, and caregivers; (3) short time completion (10–15 min maximum); (4) questions formulated from a positive life situation; (5) availability of a validated version in Spanish or Catalan, and (6) robust psychometric properties (namely reliability as well as discriminant and construct validity coefficients). The KINDL^R^ questionnaire meets all these inclusion criteria in its five versions: Kiddy-KINDL^R^ for children aged 3–6 years, Kid-KINDL^R^ for children aged 7–13 years, Kiddo-KINDL^R^ for teenagers aged 14–17 years, and the KINDL^R^ questionnaire for caregivers with children aged 3–6 or 7–17 years [69–73]. Its Spanish version shows acceptable reliability and validity coefficients and has been validated as an adequate assessment tool useful in clinical practice [74, 75].

The KINDL^R^ questionnaire consists of 24 Likert-scale standardized items associated with six QoL dimensions [68, 76]: physical well-being, emotional well-being, self-esteem, family, friends, and school (this category includes questions about daily functioning). These six dimensions can be combined to produce a total score for general well-being. Furthermore, all versions of the questionnaire contain an additional subscale named “disease,” which aims to measure children’s QoL concerning their chronic illness and hospitalization. The version of the KINDL^R^ questionnaire for caregivers of children between the ages of 3 and 6 years matches that for caregivers of children and teenagers aged 7–17 years.

The participants were asked about coping strategies through open-ended questions; these strategies were measured following the three-level hierarchical structure model of the Coping Strategies Inventory [64] based on Folkman and Lazarus’ model [77] and validated in the Spanish population [78].

### Data analysis

To analyze the data collected in the survey, we conducted descriptive analyses of the perception of QoL, well-being, and disease and of the participants’ coping strategies. We also performed comparisons between the cohorts and transformed qualitative data from coping strategies into quantitative data [79]. In the questionnaire, the participants were asked to write down the activities in which they engaged the most to cope with stressful situations. We analyzed the responses to this open-ended question following the three-level hierarchical structure model of the Coping Strategies Inventory [64] based on Folkman and Lazarus’ model [77]. This hierarchical model organizes coping strategies into two main categories: engagement and disengagement. Each category is then split into two categories: problem-focused and emotion-focused. Consequently, mid-level categories are problem-focused engagement (PFE), problem-focused disengagement (PFD), emotion-focused engagement (EFE), and emotion-focused disengagement (EFD) coping strategies. Each of these four categories is then split into two new categories, representing the final eight categories of coping strategies: problem solving (PS), which involves strategies focused on making and following a plan to overcome the faced challenge; cognitive restructuring (CR), which focuses on reappraising the situation in a way that encourages positivity; expressing emotions (EE), which are strategies focused self-disclosure of emotions and/or engagement in activities allowing people to be in contact with their internal states; social support (SS), which involves participants who turn to their social network to overcome challenges; problem avoidance (PA), which refers to focusing on certain tasks to avoid thinking about the stressful situation or conflict; wishful thinking, which expresses a desire that the situation would disappear or a miracle would happen; self-criticism (SC), which involves criticizing oneself for an event and feeling guilty; and social withdrawal (SW), which refers to avoiding time spent with others and refusing social contact. Three research members coded the responses by consensus agreement until saturation and mutual exclusion were reached [63]. Thus, we transformed the qualitative data into quantitative data [67] and analyzed the possible relationships between different coping strategies and perceptions of well-being.

The statistical analysis included testing the homogeneity of variance of KINDL scores using Levene’s test. Then, we analyzed the differences in KINDL scores for QoL and well-being and the disease variables between cohorts using Student’s t-test, one-way analysis of variance (ANOVA), two-way ANOVA, or two-way ANOVA with repeated measures adjusted for different demographic groups. When significant effects were detected in the ANOVA analysis, the Sidak post-hoc test was applied for pairwise multiple comparisons. We conducted a linear correlation study to analyze the possible relationship between well-being and disease perceptions and between the perception of well-being and the number of hospitalizations. Further, we analyzed the differences in the distribution of coping strategies between caregivers and children using Pearson’s χ^2^ test. Frequencies were presented as a percentage (%) of the total data collected; data were presented as mean ± standard error of the mean (SEM), and values of *p* < 0.05 were considered significant. We performed statistical data analyses using the statistical package SPSS Statistics v26 (IBM Corp. USA).

### Procedure

The pilot study was conducted in Catalunya, Spain between February 2021 and May 2022; however, data collection was discontinued between February 24, 2021 and January 6, 2022 due to the COVID-19 pandemic. Participants received an invitation to participate in an online survey hosted on UB Forms (University of Barcelona), which took about 15 minutes to complete. Recruitment was conducted through (1) personalized letters sent to the pediatric units of hospitals and patient associations, (2) communication with health professionals, and (3) social media dissemination (LinkedIn, Twitter, Facebook, and WhatsApp). All participants were informed about the survey’s purpose and provided their written informed consent to participate in this study; adults signed their informed consent, and children gave their assent. Recruitment of youth was addressed to parents and caregivers, who eventually gave their consent to their children’s participation in the study. Parents willing to participate received an e-mail with a link to the online questionnaire addressed to the youth, and they were also asked to help their children decide if they gave consent to participate and understand and fulfill the questionnaire. All participants were reminded that they had the right to withdraw at any time, that their participation was entirely voluntary, and that their responses would be kept confidential. The survey did not explore sensitive, private, or political information.

The project’s methods and experimental protocols were evaluated and approved on March 13, 2020 by the Ethics Committee of the University of Barcelona (Spain) before the research began (Institutional Review Board approval number-IRB00003099). The study followed the regulations established by the European Union (EU) 2016/679 of the European Parliament and of the Council from April 27 on the protection of natural persons about the processing of personal data and their free movement. It also followed the Spanish *Ley Orgánica* 3/2018, from December 5 on the protection of personal data and digital rights.

## Results

### Selection of participants meeting the inclusion criteria and final cohort

We excluded three (7.8%) surveys due to missing data, and 35 responses remained; 0% of participants answered “I don’t know/I prefer not to answer” in all analyzed variables except for “Maintain her/his hobby” in the caregiver survey, where missing data reached 8.3%. Table 2 shows the participants’ detailed demographic characteristics.

**Table 2:** Demographic characteristics of the survey participant cohort.

Characteristic	Number	%
Total number of caregivers	24	100
Female	19	79.2
Male	5	20.8
Age of the child with CMC		
3–6 years old	10	41.6
7–17 years old	14	58.4
Number of children		
One	5	20.8
More than one	19	79.2
Other relatives at home		
Yes	9	37.5
No	15	62.5
Pets at home		
Yes	17	70.8
No	7	29.2
Maintain her/his hobby		
Yes	10	41.7
No	12	50
NA	2	8.3
Child’s hospitalization status		
Hospitalized	13	54.2
Discharged	11	45.8
Total number of children / teenagers	11	100
Female	4	36.3
Male	8	72.7
Group of age		
3–6 years old	5	45.5
7–17 years old	6	54.5
Sibling at home		
Yes	11	100
No	0	-
Pets at home		
Yes	8	72.7
No	4	36.3
Hospitalized at least once		
Yes	9	81.8
No	2	18.2

NA = not answered

### Caregivers’ perception of youth’s well-being and disease

**Fig 2:**
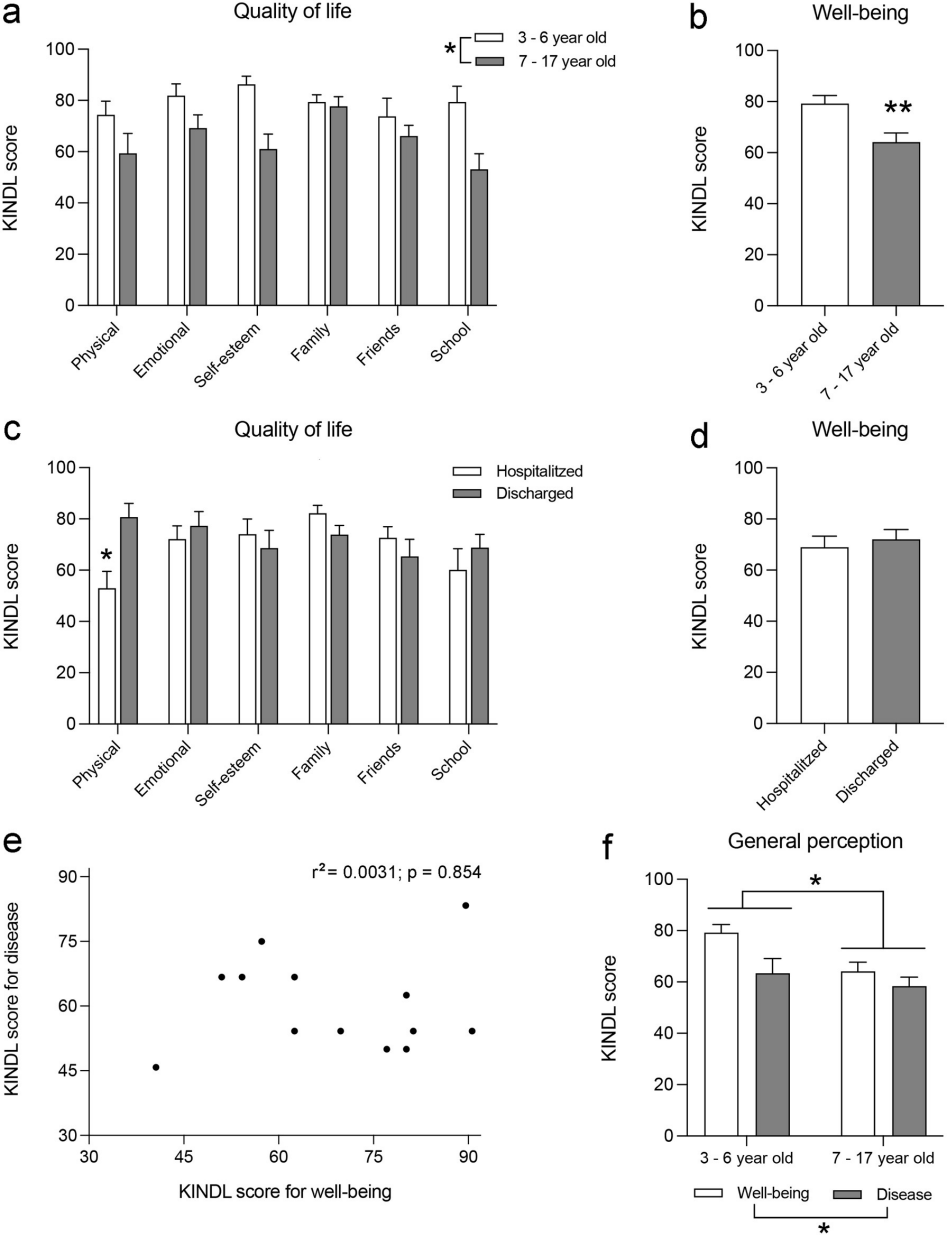
Caregivers’ perception of youth’s well-being and disease. The histograms represent the following: (a) the effects of youth’s age on the caregivers’ perception of the six dimensions of QoL defined in the KINDL^R^ questionnaire; *, *p* < 0.05, age effect, two-way ANOVA; (b) the effect of youth’s age on caregivers’ perception of youth’s general well-being; **, *p* < 0.01, different from 3 to 6 years, Student’s t-test; (c) effect of the youth’s hospitalization situation on the caregivers’ perception of the six dimensions of QoL defined in the KINDL^R^ questionnaire; *, *p* < 0.05, different from physical well-being with hospital discharge, Sidak post-hoc test; (d) the effect of the youth’s hospitalization situation on the caregivers’ perception of the youth’s general well-being; (e) linear correlation analysis between the caregivers’ perception of well-being and that of children’s disease; (f) comparison of the caregivers’ perception of well-being and disease depending on the age group of their children; *, *p* < 0.05, two-way ANOVA. The histograms present the mean ± SEM.

The caregivers’ KINDL score for the perception of their children’s general well-being was 70.40 ± 2.88. We analyzed the differences in the perception of the six dimensions of QoL by the youth’s age groups. In the two-way ANOVA with repeated measures, we found a significant effect of age, with F_(1,13)_ = 8.130; *p* = 0.0128, but no effect of the QoL dimension or interaction between both parameters, with F_(5,65)_ = 2.222; *p* = 0.0626 and F_(5,41)_ = 2.133; *p* = 0.0807, respectively. Therefore, although certain trends are visible, we detected no significant differences between the different dimensions of youth’s QoL as perceived by caregivers. Conversely, caregivers of children aged 3–6 years had a higher perception of QoL in most of its dimensions than the caregivers of youth aged 7–17 years (Fig 2a). This difference was also evident when the KINDL scores for general well-being were compared: the score was 23.4% higher among caregivers of children aged 3–6 years (t = 2.981; *p* = 0.0069; Fig 2b).

Then, we analyzed the effect of hospitalization on the KINDL scores of QoL that caregivers gave to their children. A two-way ANOVA with repeated measures revealed significant differences between QoL dimensions, F_(5,60)_ = 2.826; *p* = 0.0234; without a significant effect of hospitalization status, F_(1,12)_ = 0.090; *p* = 0.7689; but with a strong interaction between both parameters, F_(5,48)_ = 5.345; *p* = 0.0006. According to the post-hoc analysis, physical well-being had a lower KINDL score compared with the other dimensions (Fig 2c). In addition, the interaction between both parameters indicated that the group of caregivers of hospitalized youth is responsible for this decrease (Fig 2c). When analyzing the KINDL score for general well-being, the effect of physical well-being was offset by the other dimensions, and no differences were observed when comparing the scores given by the caregivers of hospitalized and discharged youth (t = 0.0523; *p* = 0.6056; Fig 2d).

Among the participants, 13 caregivers stated that their children were hospitalized, and answered the KINDL questions about disease perception with a mean value of 56.30 ± 4.79, which was 25% lower than the score obtained for the caregivers’ perception of youth’s well-being (t = 2.504; *p* = 0.0123). Then, we analyzed the effect of the children’s age on the differences between well-being and disease perceptions. The two-way ANOVA showed differences between the perceptions of well-being and disease of caregivers, F_(1,33)_ = 5.754; *p* = 0.0223, and also a significant effect of age, F_(1,33)_ = 6.705; *p* = 0.0142, but no interaction between both parameters, F_(1,33)_ = 1.449; *p* = 0.2372. This indicates that caregivers of children aged 3–6 years had higher perceptions of both the well-being and disease of their children than those of youth aged 7–17 years (Fig 2f). In particular, we performed a correlation study between the perception of well-being and the perception of disease presented by the caregivers of hospitalized youth and found no linear relationship between both parameters (r^2^ = 0.0031; *p* = 0.854), which suggests that caregivers’ perceptions of well-being and disease are independent, without a clear relationship between them (Fig 2e).

Finally, we performed a demographic study and analyzed the caregivers KINDL scores for well-being and disease based on whether their children with CMC had siblings, lived with other family members, or had a pet at home, and whether caregivers maintained their hobbies (Table 3). We found no differences in the perception of well-being among caregivers depending on whether the children had siblings; however, we detected a greater perception of youth’s well-being among caregivers who lived without other relatives at home, those who did not have pets at home, and those who kept their hobbies (Table 3). In all these situations, we found no significant differences in the caregivers’ perception of the youth’s disease (Table 3).

**Table 3:** Demographic analysis of the caregivers’ perception of children’s well-being and disease.

	Well-being			Disease		
	KINDL score	t-test	p-value	KINDL score	t-test	p-value
Siblings						
Yes	68.65 ± 3.36	1.199	0.2433	70.28 ± 2.77	0.037	0.9708
No	77.08 ± 4.69			70.46 ± 3.20		
Relatives at home						
Yes	62.39 ± 4.05	2.357	0.0278*	68.95 ± 2.33	0.439	0.6699
No	75.21 ± 3.43			71.13 ± 3.24		
Pets at home						
Yes	66.74 ± 3.45	2.130	0.0446*	69.53 ± 3.35	0.532	0.6061
No	79.31 ± 3.61			72.15 ± 1.18		
Caregivers keep hobbies						
Yes	82.09 ± 2.38	5.780	<0.0001***	73.87 ± 2.68	1.661	0.1276
No	59.47 ± 2.96			66.93 ± 3.19		

KINDL score = mean ± SEM; two tailed t-test;

* *p* < 0.05;

*** *p* < 0.0001

### Youth’s perception of their own well-being and disease

**Fig 3:**
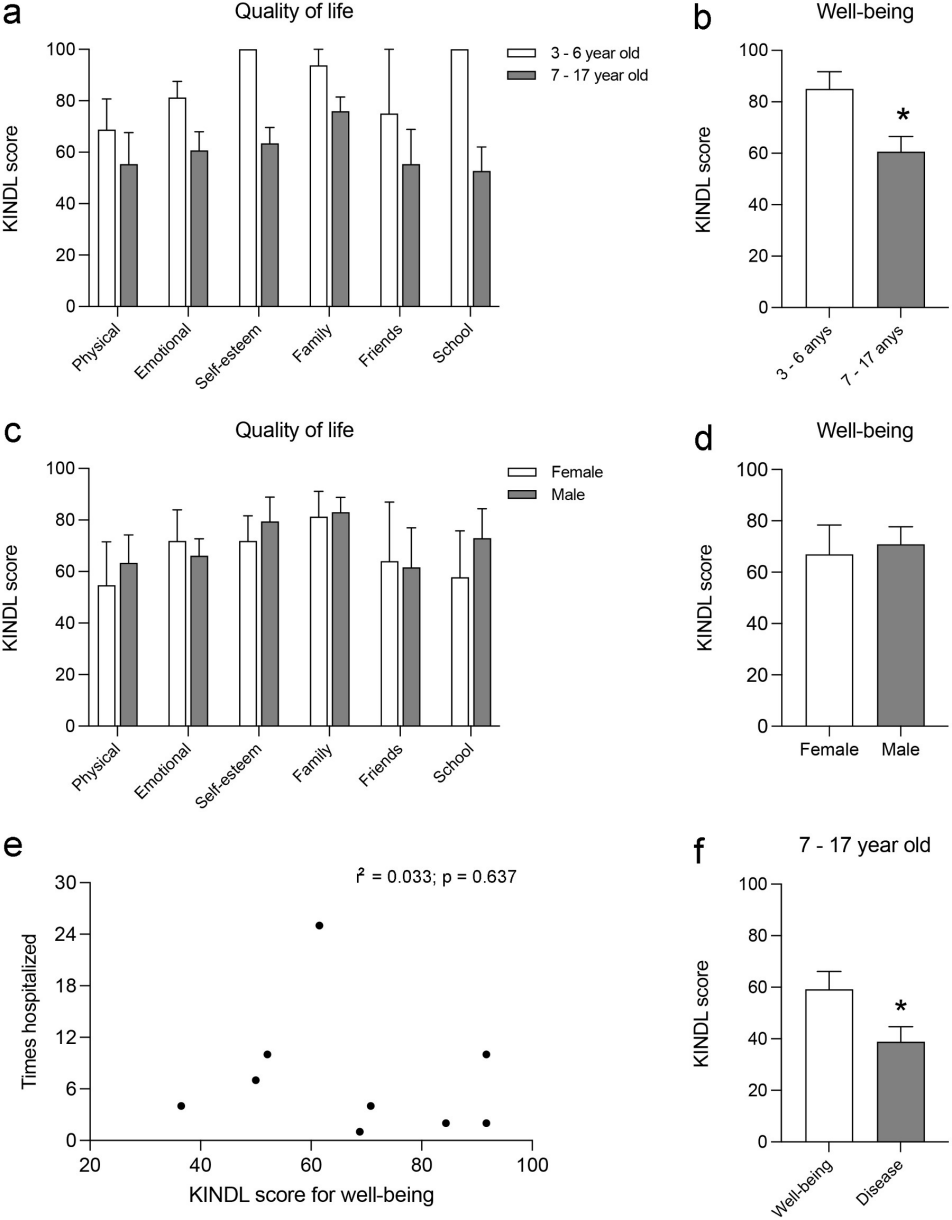
Youth’s perception of their own well-being and disease. The histograms represent the following: (a) the effect of age on the perception of the six dimensions of QoL defined in the KINDL^R^ questionnaire; (b) the effect of youth’s age on their own perception of general well-being; *, *p* < 0.05, different from 3 to 6 years, Student’s t-test; (c) the effect of gender on the perception of the six dimensions of QoL defined in the KINDL^R^ questionnaire; (d) the effect of gender on the perception of general well-being; (e) linear correlation analysis between the youth’s perception of well-being and the number of hospitalizations; (f) comparison of the perceptions of well-being and disease of the hospitalized 7–17-year-old youth; *, *p* < 0.05, different from well-being, Student’s t-test. The histograms present the mean ± SEM.

We analyzed the differences in the perception of the six QoL dimensions by the youth’s age groups, and in the two-way ANOVA with repeated measures, we found no significant effect of age, F_(1,6)_ = 3.103; *p* = 0.1286; QoL dimensions, F_(5,30)_ = 1.870; *p* = 0.1293; or interaction between the two parameters, F_(5,11)_ = 0.701; *p* = 0.6341 (Fig 3a). However, when the KINDL scores for general well-being were compared, we observed this score being 28.7% smaller in the group of youth aged 7–17 (t = 2.591; *p* = 0.0292; Fig 3b). The KINDL score indicating the perception of own well-being of the 11 youth participating in the study gave an average value of 69.5 ± 5.9.

We also analyzed the possible differences in QoL perception based on the youth’s gender. In the two-way ANOVA with repeated measures, we found no significant effect of gender, F_(1,6)_ = 0.075; *p* = 0.7930; of the QoL dimensions, F_(5,30)_ = 1.307; *p* = 0.2847; or of the interaction between both parameters, F_(5,11)_ = 0.284; *p* = 0.9117 (Fig 3c). Furthermore, we also found no differences when comparing the general well-being scores between females and males (t = 0.319; *p* = 0.7564; Fig 3d).

Next, we analyzed the effect of hospitalization on the youth’s perception of their own QoL and well-being. To estimate the possible influence of hospitalizations on youth’s well-being, we conducted a correlation analysis between their perception of well-being and the number of hospitalizations they had experienced. We did not find a linear correlation between the parameters (r^2^ = 0.033; *p* = 0.6737; Fig 3e), which indicates that, under our conditions, the youth’s perception of well-being did not have a clear linear relationship with the number of hospitalizations they experienced. The KINDL^R^ questionnaire for children aged 3–6 years has no disease section; consequently, we compared perceptions of well-being and disease only in hospitalized youth aged 7–17 (Fig 3f). In this group, the KINDL score for the disease perception gave an average value of 38.88 ± 5.82, which is 33.1% smaller than the score obtained for the perception of well-being in the same age group (59.22 ± 6.89; t = 3.061; *p* = 0.0376).

Finally, given that 93% of youth with CMC participating in the study had siblings, we could not estimate the effect that this presence had on their perception of well-being. We analyzed the effect of the presence of pets at home and found no influence on this perception (KINDL score 71.04 ± 7.16 for pets at home and 65.30 ± 10.11 for no pets at home; t = 0.430; *p* = 0.6769).

### Comparison of youth’s perceptions of their well-being and disease with the caregivers’ perceptions

**Fig 4:**
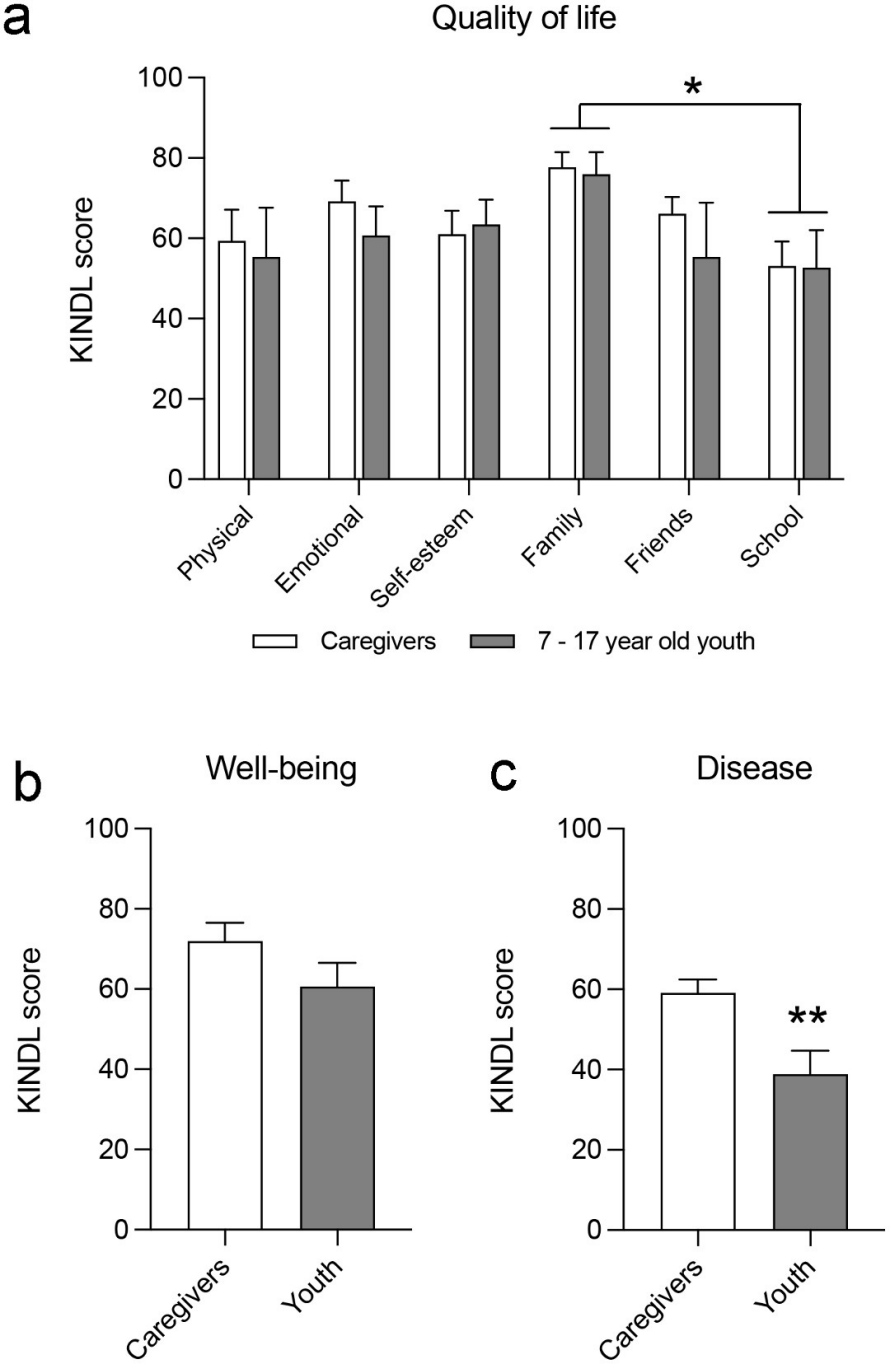
Comparison of youth’s perceptions of their well-being and disease with the caregivers’ perceptions. The histograms represent (a) a comparison of the perception of the six QoL dimensions defined in the KINDL^R^ questionnaire between caregivers and youth aged 7–17 years; *, *p* < 0.05, Sidak post-hoc; a comparison of the perception of general well-being (b) and disease (c) between caregivers and hospitalized youth aged 7–17; *, *p* < 0.05 different from caregivers, Student’s t-test. Histograms show the mean ± SEM.

The version of the KINDL^R^ questionnaire for children aged 3–6 years differs from those of the other youth groups in the number of items and type of scale. However, the versions of the questionnaire for youth aged 7–17 years and for caregivers quantify the perception of QoL, well-being, and disease equally. Therefore, we made a comparative analysis between the KINDL scores of youth aged 7–17 answering the questionnaire (n = 7) and the scores of caregivers of youth of the same age (n = 12). We first compared the KINDL scores of both groups in each of the six QoL dimensions (Fig 4a). The two-way ANOVA analysis with repeated measures showed no differences between the perception of youth and that of caregivers, F_(1,19)_ = 0.330; *p* = 0.572, but it showed a significant effect of the QoL dimension, F_(5,95)_ = 3.543; *p* = 0.006, without interaction between both parameters, F_(5,95)_ = 0.329; *p* = 0.894. This indicates that youth have a perception of their own well-being similar to that of their caregivers (Fig 4a). In addition, both groups present differences in their perceptions of the QoL dimensions. Specifically, the family dimension had the highest KINDL score (77.11 ± 4.47); this was significantly greater than that of the school dimension (*p* = 0.021; Sidak post-hoc), which comparatively presented the lowest score (52.99 ± 5.08).

Subsequently, we compared the perceptions of general well-being and disease presented by hospitalized youth aged 7–17 (n = 5) with those of caregivers of youth of the same age (n = 11). Under our conditions, we did not observe differences when comparing the general well-being scores between both groups (t = 1.529; *p* = 0.1457) (Fig 4b); on the contrary, we found that the perception of disease in youth was 34.2% lower than that detected in caregivers (t = 3.228; *p* = 0.0061; Fig 4c).

### Relationship of well-being perception and coping strategies

**Fig 5:**
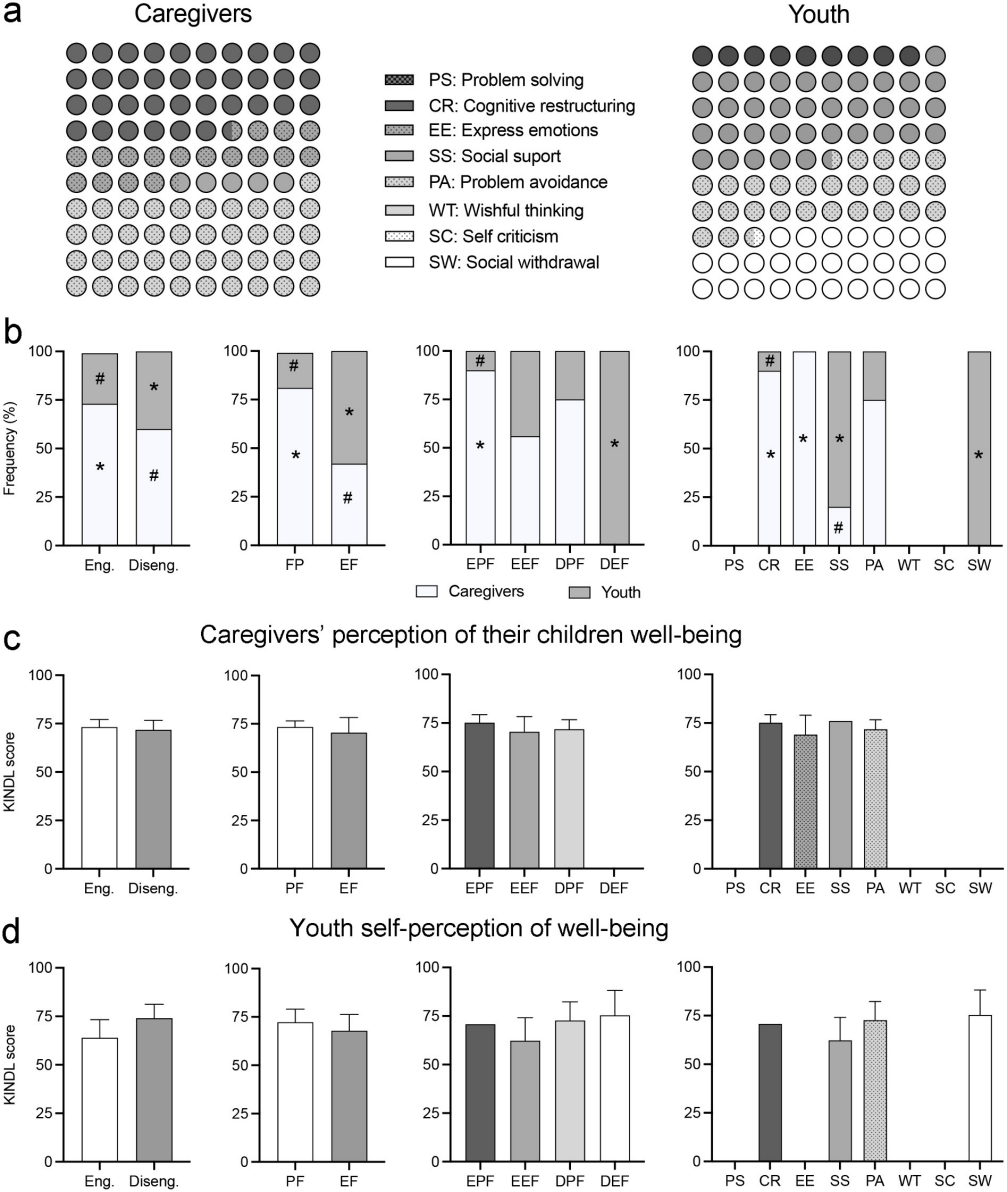
Relationship of well-being perception with coping strategies. Analysis of the relationship of well-being perception with the coping strategies displayed to deal with stressful situations. (a) Graphical representation of the percentage of participants in each coping strategy category (see text for details). (b) Histograms of the contingency tables showing comparisons of coping strategies by group of participants; *, *p* < 0.05 values greater than expected, #, *p* < 0.05, values smaller than expected, Pearson’s χ^2^. (c) Histograms show the KINDL score of the perception that caregivers have of their children’s well-being for every category of coping strategy. (d) Histograms show the KINDL score of the youth’s perception of well-being for every category of coping strategy. Eng. = engagement, Diseng. = disengagement; PF = problem-focused; EE = emotion-focused; PFE = problem-focused engagement; EFE = emotion-focused engagement; PFD = problem-focused disengagement; EFD = emotion-focused disengagement.

All participants in the study showed various coping strategies to deal with stressful situations. Most of the strategies reported by caregivers belonged to the PA and CR categories (40.9% and 36.4% of the total, respectively), while EE strategies were reported in 18.2% of cases and SS in 4.5%. Interestingly, caregivers engaged in very few EFD activities, with no SC and SW strategies reported (Fig 5a). Coping strategies used by youth differed from those of caregivers: SS was greatly used by 36.4% of youth, followed by SW and PA strategies, each with a frequency of 27.3% (Fig 5a); finally, 9.1% engaged in CR, and 0% reported PS and SC strategies.

We compared the frequencies of the strategies reported by caregivers and youth and found statistically significant differences in the frequency distribution of the groups (χ^2^ = 51.421, *p* < 0.0001; Fig 5b). Among all the differences in distribution, only caregivers reported EE strategies, and only youth displayed SW strategies. In addition, youth reported adopting SS strategies more frequently, while caregivers adopted CR strategies (Fig 5b). When we analyzed the frequency of coping strategies classified by secondary and tertiary scales, we also observed differences. Thus, youth displayed emotion-focused strategies (χ^2^ = 40.888, *p* < 0.0001) and disengagement strategies (χ^2^ = 35.738, *p* < 0.0001) more frequently than caregivers, who mostly displayed problem-focused strategies (Fig 5b); consequently, EFD and PFE strategies were more frequent among youth than among caregivers (χ^2^ = 44.731, *p* < 0, 0001).

Then, we analyzed the possible differences in the well-being KINDL score of youth associated with coping strategies. The one-way ANOVA showed no differences in the KINDL scores of caregivers’ perception of the youth’s well-being in any of the coping strategy classification levels (Fig 5b); thus, we found no differences in the mean KINDL score between coping strategies when classified into eight categories, F_(3,19)_ = 0.184, *p* = 0.906. This was also true for either the comparison between engagement and disengagement strategies, F_(1,19)_ = 0.214, *p* = 0.809; the comparison of emotion-focused vs. problem-focused strategies, F_(1,21)_ = 0.271, *p* = 0.766; or middle-level classification, F_(3,18)_ = 0.242, *p* = 0.866. Similar results were obtained when we analyzed the effect of coping strategy on the youth’s perception of their own well-being. We observed no effect of the coping category in the mean KINDL scores—F_(3,10)_ = 0.251, *p* = 0.859—of the eight coping strategies: F_(1,9)_ = 0.758, *p* = 0.406 for the disengagement vs. disengagement classification; F_(1,10)_ = 0.124, *p* = 0.733 for emotion-focused vs. problem-focused classification; and F_(3,10)_ = 0.251, *p* = 0.859 for middle-level classification (Fig 5c).

## Discussion

This pilot study compared the perception of children and adolescents with CMC in the situation of hospitalization and convalescence and that of their caregivers. Our results support the beneficial effects of the family environment on youth’s QoL; the main findings, in particular, support the independent relationship between caregivers’ perceptions of well-being and disease. In addition, the perception of well-being in children with a CMC impacts the way they view, relate, and communicate with their immediate environment.

Regarding QoL perception, we found no significant differences between the QoL perceived by youth and that perceived by their caregivers, which means that caregivers view youth’s QoL according to how they view themselves. This is a highly positive finding as research has previously shown that parental catastrophizing leads to parental overprotection [80], higher child’s disability and low school attendance [81, 82], and restriction of their children’s ability [83]. Caregivers of children aged 3–6 years had a higher perception of well-being in most of its dimensions than caregivers of youth aged 7–17 years, and these lower levels in the older cohort are also found in the measures of youth’s perceived own well-being. A possible explanation to this finding is that as children become adolescents, youth’s limitations due to their illness become more obvious. This interpretation could also explain why physical well-being is reported as the lowest out of all other dimensions. Nevertheless, we did not find a correlation between the caregiver’s perceptions of well-being and disease.

Furthermore, we found no differences in the caregivers’ perception of well-being depending on whether the children had siblings; however, we detected a greater perception of youth’s well-being in caregivers who lived without other relatives at home, those who did not have pets at home, and those who kept their hobbies. This could be the result of added stress as the household dynamics get more complex: a pet adds another dependent entity, and relatives at home may be an added financial burden, especially if they are grandparents who need care. Three-generational households may strengthen perceived social support, but the positive effects may be dependent on the quality of the relationships and the financial burden [84].

When we explored coping strategies, we found that youth coped with stressful situations by engaging in the social support category, followed by strategies of the social withdrawal and problem avoidance categories. Interestingly, no youth reported strategies in the express emotions category. Types of coping strategies can influence the HRQoL of youth with CMC. For example, in a previous study, adaptive coping styles and social support were negatively correlated with psychological distress and anxiety in children with malignant tumors [85]. Additionally, we found no clear relationship between the youth’s perception of well-being and the coping strategies displayed. However, some tendencies were observed as youth displaying strategies of emotion-focused engagement—particularly social support—presented slightly lower well-being scores than youth presenting problem-focused disengagement, particularly problem avoidance. These results align with the previous literature reporting that children with CMC who express emotional reactions are negatively related to HRQoL [86, 87], while acceptance and avoidance are positively correlated [87, 88].

We found substantial differences between caregivers and youth in the coping strategies used to deal with stressful situations. Most of the strategies that caregivers engaged in belonged to the problem avoidance, cognitive restructuring, and—to a lesser extent—express emotions categories, while barely reporting emotion-focused disengagement strategies, which indicates that in our sample, caregivers preferred to display problem-focused strategies that individually manage the stressful situation than emotion-focused strategies that involve receiving understanding and comfort from others. Previous studies support this finding (e.g., [89]). Although this preference for problem-focused strategies may be associated with lower well-being outcomes [90], studies have reported contradictory effects of coping styles on caregivers’ psychological distress. For example, increased social support has been associated with lower caregiver motivation in parents of youth with type 1 diabetes [91]; in contrast, emotional support has also been associated with the well-being improvement of caregivers of children with rare diseases [92]. Indeed, fostering caregivers’ social support and cognitive restructuring strategies may be relevant in increasing parenting self-efficacy to successfully address children’s illnesses by helping them in emotion management [93]. As increased parental self-efficacy in health contexts has been reported to improve caregivers’ confidence and self-esteem [94], cognitive restructuring may constitute an adaptive coping strategy for improving caregivers’ well-being.

The differences between coping styles displayed by caregivers and youth emphasize that they do not tend to cope with stress jointly, which is an important finding because the way in which youth and caregivers deal with a CMC is not only affected by disease-related stress but also by stress perceived from each other [95]. Children’s low emotional functioning particularly affects the psychosocial aspects of the caregivers’ QoL, which in turn determines the way that the child confronts the CMC. Indeed, family-based support and affection are derived from QoL and well-being [96]. In addition, reinforcement of the bonding integration and connectivity between family members can enhance QoL [97]; thus, dyadic coping [98] emerges as an important agent to deal with the CMC-related stress of both youth and caregivers, although it has still been poorly studied in child–caregiver relationships. However, a study reported that emotion-oriented dyadic coping has been associated with HRQoL in children with CMC [95]. Moreover, children’s physical well-being has been linked to the caregivers’ use of acceptance coping strategies [86] commonly displayed by caregivers in our study (cognitive restructuring strategies). However, the same study reports that this acceptance coping is not positively linked to the caregivers’ own psychosocial well-being [86]. Nevertheless, communication between youths and caregivers appears crucial in understanding each other’s feelings and in providing appropriate dyadic coping with positive effects on the children’s HRQoL and well-being. Furthermore, helping children and caregivers to jointly deal with CMC-related stressful situations may not only empower youth and benefit their HRQoL, but it may also improve the caregivers’ QoL.

### Limitations and future directions

This pilot study has certain limitations. First, sampling was non-randomized with the convenience selection criterium. Moreover, as recruitment was discontinued due to COVID-19 restrictions, the sample was small, which lessened the power of the statistical analysis of certain parameters, such as the illness perception of children aged 3–6 years and the frequency of some coping strategies. In the analysis of these parameters, the study’s statistical power only reached 29.5%, which may mask possible differences and crossover effects between the groups. Nonetheless, the sample was sufficient to achieve statistically significant results (25% effect with a significance level of 0.05) in the perception of well-being and identification of coping strategies. In this line, this pilot study helps to establish the sample size and characteristics for further larger studies analyzing the HRQoL of children with CMC. Moreover, as the surveys were accessed online, families without reliable access to the Internet or an electronic device may have been missed. Finally, self-reported responses may have been impacted by a poor view of the participants’ internal states; however, using a survey tool to measure well-being and psychological distress is a common practice in this field.

The understanding of hospitalization and care processes constitutes a barrier for the QoL and well-being of youth with CMC and their caregivers as the quantity of complex information to assimilate is overwhelming [99–101]. Personalized actions to improve communication with youth with CMC and with their caregivers are necessary to improve their perception of family well-being and help them display adaptive coping strategies [102, 103]. The literature includes various studies addressing the impact of hospitalization in CMC from the youth’s point of view (e.g., [104]) on their needs (e.g., [105]) and supportive care (e.g., [106]). However, a gap still exists with regard to some concrete issues, such as hospitalization and readmission [107] or isolation [104], and the same is true for suffering, spiritual pain, or emotional stress regarding further procedures—especially those involving needle procedures [108]. Future studies should conduct comparative longitudinal studies with and without intervention-related parenting and child- and family-centered care, which would allow to see the features that improve QoL and well-being in the family group.

## Conclusions

Our findings show the paramount importance of evaluating the bidirectional impact of the experiences of youth with CMC and their support system; furthermore, they highlight the consequences that such dynamics have on the QoL of this youth. So far, little research has asked youth to participate directly, and investigations have mostly revolved around health professionals and caregivers. This preliminary study shows the importance of directly exploring the experience of youth as well as facilitating communication spaces with both the family and health professionals where the voice of children is considered. In addition, coping strategies differ between caregivers and youth; therefore, mental health professionals should recognize these differences and work accordingly. Finally, policymakers should integrate these findings when developing and implementing guidelines around the best practices of pediatric care. Our findings recognize and reiterate the importance of listening to the voice of children and adolescents and their families regarding pediatric care practices. We hope that this pilot will spearhead the development of further research exploring the experiences of children in the same way that certain research projects have been doing with parental experiences [109]. The literature in this field can positively impact the way health professionals care for patients and the quality of the healthcare system.

## Supporting information

S1 Data (XLSX)

S2 Data (ZIP)

## References

[1] Burns KH, Casey PH, Lyle RE, Bird T Mac, Fussell JJ, Robbins JM. Increasing prevalence of medically complex children in US hospitals. Pediatrics. 2010;126(4):638–46. doi: 10.1542/peds.2009-1658 20855383

[2] Vieira MA, de Lima RAG. Crianças e Adolescentes com doença crônica: convivendo com mudanças. Rev Latino-am Enferm. 2002;10(4):552–60. 12592857

[3] Cohen E, Kuo DZ, Agrawal R, Berry JG, Bhagat SKM, Simon TD, et al. Children with medical complexity: An emerging population for clinical and research initiatives. Pediatrics. 2011;127(3):529–38. doi: 10.1542/peds.2010-0910 21339266 PMC3387912

[4] McPherson M, Arango P, Fox H, Lauver C, Mcmanus M, Newacheck PW, et al. A New Definition of Children. Pediatrics. 1998;102(1):137–40. 9714637 10.1542/peds.102.1.137

[5] Barrio Cortes J, Suárez Fernández C, Bandeira de Oliveira M, Muñoz Lagos C, Beca Martínez MT, Lozano Hernández C, et al. Chronic diseases in the paediatric population: Comorbidities and use of primary care services. An Pediatr. 2020;93(3):183–93. 10.1016/j.anpedi.2019.12.019 32178966

[6] Climent Alcalá FJ, García Fernández de Villalta M, Escosa García L, Rodríguez Alonso A, Albajara Velasco LA. Unidad de niños con patología crónica compleja. Un modelo necesario en nuestros hospitales. An Pediatría. 2018;88(1):12–8. 10.1016/j.anpedi.2017.04.002 28499736

[7] Berg Kelly K. Sustainable transition process for young people with chronic conditions: A narrative summary on achieved cooperation between paediatric and adult medical teams. Child Care Health Dev. 2011;37(6):800–5. doi: 10.1111/j.1365-2214.2011.01330.x 22007979

[8] Berry JG, Hall M, Cohen E, O’Neill M, Feudtner C. Ways to Identify Children with Medical Complexity and the Importance of Why. J Pediatr. 2015;167(2):229–37. doi: 10.1016/j.jpeds.2015.04.068 26028285 PMC5164919

[9] Dewan T, Cohen E. Children with medical complexity in Canada. Paediatr Child Heal. 2013;18(10):518–22. doi: 10.1093/pch/18.10.518 24497777 PMC3907346

[10] Moreira MCN, Albernaz LV, de Sá MRC, Correia RF, Tanabe RF. Recomendações para uma linha de cuidados para crianças e adolescentes com condições crônicas complexas de saúde. Cad Saude Publica. 2017;33(11). 10.1590/0102-311X00189516 29166490

[11] Srivastava R, Stone BL, Murphy NA. Hospitalist care of the medically complex child. Pediatr Clin North Am. 2005;52(4):1165–87. doi: 10.1016/j.pcl.2005.03.007 16009262

[12] Typpo K V., Petersen NJ, Petersen LA, Mariscalco MM. Children with Chronic Illness Return to Their Baseline Functional Status after Organ Dysfunction on the First Day of Admission in the Pediatric Intensive Care Unit. J Pediatr [Internet]. 2010;157(1):108–113.e1. Available from: doi: 10.1016/j.jpeds.2009.12.029 20223474 PMC4406357

[13] Yu JA, Henderson C, Cook S, Ray K. Family Caregivers of Children With Medical Complexity: Health-Related Quality of Life and Experiences of Care Coordination. Acad Pediatr [Internet]. 2020;20(8):1116–23. Available from: doi: 10.1016/j.acap.2020.06.014 32599346 PMC8063607

[14] Pinquart M. Parenting stress in caregivers of children with chronic physical condition—A meta-analysis. Stress Heal. 2018;34(2):197–207. doi: 10.1002/smi.2780 28834111

[15] Ahmadi B, Sabery M, Adib-Hajbaghery M. Burnout in the Primary Caregivers of Children With Chronic Conditions and its Related Factors. J Client-centered Nurs Care. 2021;7(2):139–48.

[16] Nabors L, Liddle M. Perceptions of Hospitalization by Children with Chronic Illnesses and Siblings. J Child Fam Stud [Internet]. 2017;26(6):1681–91. Available from: doi: 10.1007/s10826-017-0688-6

[17] Delvecchio E, Salcuni S, Lis A, Germani A, Di Riso D. Hospitalized Children: Anxiety, Coping Strategies, and Pretend Play. Front Public Heal. 2019;7(September):1–8. doi: 10.3389/fpubh.2019.00250 31555632 PMC6743064

[18] Bergmans RS, Smith J. Associations of mental health and chronic physical illness during childhood with major depression in later life. Aging Ment Health [Internet]. 2022 Sep 2;26(9):1813–20. Available from: doi: 10.1080/13607863.2021.1958143 34353181 PMC8818054

[19] Alfaro Rojas AK, Atria Machuca RP. Factores ambientales y su incidencia en la experiencia emocional del niño hospitalizado. Rev Pediatría Electrónica [Internet]. 2009;6(1):36–54. Available from: http://www.revistapediatria.cl/volumenes/2009/vol6num1/pdf/FACTORES_AMBIENTALES.pdf

[20] Doupnik SK, Henry MK, Bae H, Litman J, Turner S, Scharko AM, et al. Mental Health Conditions and Symptoms in Pediatric Hospitalizations: A Single-Center Point Prevalence Study. Acad Pediatr. 2017;17(2):184–90. doi: 10.1016/j.acap.2016.08.009 28259340

[21] Kaminski L, Joyce KM, Simpson KM, Hons BA, Wittmeier K, Benzies K, et al. A Scoping Review of Mental Health Programs for Parents of Children with Complex Medical Conditions. PsyArXiv Novemb 19. 2021;1–36.

[22] Lum A, Wakefield CE, Donnan B, Burns MA, Fardell JE, Jaffe A, et al. School students with chronic illness have unmet academic, social, and emotional school needs. Sch Psychol. 2019;34(6):627–36. doi: 10.1037/spq0000311 31697148

[23] Matthew P. Myrvik MP, Campbell, Andrew D, Davis MM, Butcher JL. Impact of Psychiatric Diagnoses on Hospital Length of Stay in Children With Sickle Cell Anemia. Pediatr Blood Cancer. 2012;58(February):239–43. 21425450 10.1002/pbc.23117

[24] Myrvik MP, Burks Lisa M, Hoffman Raymond G, Dasgupta M, Panepinto Julie A. Mental Health Disorders influence admission Rates for Pain in Children with Sickle Cell Disease. Pediatr Blood Cancer. 2013;(February):1388–9. doi: 10.1002/pbc.24394 23151972

[25] Mevorach T, Taler M, Dar S, Lebow M, Sapir IS, Rotkopf R, et al. The relationship between the plasma proinflammatory cytokine levels of depressed/anxious children and their parents. Sci Rep [Internet]. 2021;11(1):1–9. Available from: doi: 10.1038/s41598-021-90971-4 34083584 PMC8175361

[26] Lee J, Chi S, Lee MS. Molecular biomarkers for pediatric depressive disorders: A narrative review. Int J Mol Sci. 2021;22(18). doi: 10.3390/ijms221810051 34576215 PMC8464852

[27] Foster K, Young A, Mitchell R, Van C, Curtis K. Experiences and needs of parents of critically injured children during the acute hospital phase: A qualitative investigation. Injury [Internet]. 2017;48(1):114–20. Available from: doi: 10.1016/j.injury.2016.09.034 27692666

[28] Kolaitis G, Giannakopoulos G, Liakopoulou M, Pervanidou P, Charitaki S, Mihas C, et al. Predicting pediatric posttraumatic stress disorder after road traffic accidents: The role of parental psychopathology. J Trauma Stress [Internet]. 2011 Aug;24(4):414–21. Available from: https://onlinelibrary.wiley.com/doi/10.1002/jts.20667 21812037 10.1002/jts.20667

[29] Muscara F, McCarthy MC, Thompson EJ, Heaney CM, Hearps SJC, Rayner M, et al. Psychosocial, Demographic, and Illness-Related Factors Associated With Acute Traumatic Stress Responses in Parents of Children With a Serious Illness or Injury. J Trauma Stress. 2017;30(3):237–44. doi: 10.1002/jts.22193 28644537

[30] Arabzadeh M, Tirgari B, Farokhzadian J, Mohammadalizadeh S. Family environment, parental stressors, and post-traumatic stress disorder in the parents of premature infants in the Neonatal Intensive Care Unit. J Pediatr Neonatal Individ Med. 2022;11(2):1–13.

[31] Cohn LN, Pechlivanoglou P, Lee Y, Mahant S, Orkin J, Marson A, et al. Health Outcomes of Parents of Children with Chronic Illness: A Systematic Review and Meta-Analysis. J Pediatr [Internet]. 2020;218:166–177.e2. Available from: doi: 10.1016/j.jpeds.2019.10.068 31916997

[32] Malin KJ, Johnson TS, Mcandrew S, Westerdahl J, Leuthner J, Lagatta J. Infant illness severity and perinatal post-traumatic stress disorder after discharge from the neonatal intensive care unit Kathryn. 2021; 10.1016/j.earlhumdev.2019.104930 PMC7237277 31759276

[33] Van Oers HA, Haverman L, Limperg PF, Van Dijk-Lokkart EM, Maurice-Stam H, Grootenhuis MA. Anxiety and Depression in Mothers and Fathers of a Chronically Ill Child. Matern Child Health J. 2014;18(8):1993–2002. doi: 10.1007/s10995-014-1445-8 24791971

[34] Bayat M, Erdem E, Gül Kuzucu E. Depression, anxiety, hopelessness, and social support levels of the parents of children with cancer. J Pediatr Oncol Nurs. 2008;25(5):247–53. doi: 10.1177/1043454208321139 18648089

[35] McCarthy MC, Ashley DM, Lee KJ, Anderson VA. Predictors of Acute and Posttraumatic Stress Symptoms in Parents Following Their Child’s Cancer Diagnosis. J Trauma Stress [Internet]. 2012 Oct;25(5):558–66. Available from: https://onlinelibrary.wiley.com/doi/10.1002/jts.21745 23055298 10.1002/jts.21745

[36] Ozdemir Koyu H, Tas Arslan F. The effect of physical and psychosocial symptoms on caregiver burden of parents of children with cancer. Eur J Cancer Care (Engl). 2021;30(6):1–11. doi: 10.1111/ecc.13513 34632650

[37] Doherty N, McCusker CG, Molloy B, Mulholland C, Rooney N, Craig B, et al. Predictors of psychological functioning in mothers and fathers of infants born with severe congenital heart disease. J Reprod Infant Psychol. 2009;27(4):390–400.

[38] Gregory MRB, Prouhet PM, Russell CL, Pfannenstiel BR. Quality of Life for Parents of Children with Congenital Heart Defect: A Systematic Review. J Cardiovasc Nurs. 2018;33(4):363–71. doi: 10.1097/JCN.0000000000000466 29601369

[39] Mussatto KA, Van Rompay MI, Trachtenberg FL, Pemberton V, Young-Borkowski L, Uzark K, et al. Family Function, Quality of Life, and Well-Being in Parents of Infants With Hypoplastic Left Heart Syndrome. J Fam Nurs. 2021;27(3):222–34. doi: 10.1177/1074840720987309 33535863 PMC8594631

[40] Solberg Ø, Grønning Dale MT, Holmstrøm H, Eskedal LT, Landolt MA, Vollrath ME. Long-term symptoms of depression and anxiety in mothers of infants with congenital heart defects. J Pediatr Psychol. 2011;36(2):179–87. doi: 10.1093/jpepsy/jsq054 20558484 PMC3042598

[41] Logan GE, Sahrmann JM, Gu H, Hartman ME. Parental Mental Health Care After Their Child’s Pediatric Intensive Care Hospitalization*. Pediatr Crit Care Med [Internet]. 2020 Nov 18;21(11):941–8. Available from: https://journals.lww.com/10.1097/PCC.0000000000002559 32947380 10.1097/PCC.0000000000002559 PMC7609586

[42] Riley AR, Williams CN, Moyer D, Bradbury K, Leonard S, Turner E, et al. Parental Posttraumatic Stress Symptoms in the Context of Pediatric Post-Intensive Care Syndrome: Impact on the Family and Opportunities for Intervention. Clin Pract Pediatr Psychol. 2021;9(2):156–66. doi: 10.1037/cpp0000399 34458053 PMC8386200

[43] Yagiela LM, Carlton EF, Meert KL, Odetola FO, Cousino MK. Parent Medical Traumatic Stress and Associated Family Outcomes after Pediatric Critical Illness: A Systematic Review. Pediatr Crit Care Med. 2019;20(8):759–68. doi: 10.1097/PCC.0000000000001985 31107380

[44] Wiseman T, Curtis K, Young A, Van C, Foster K. ‘It’s turned our world upside down’: Support needs of parents of critically injured children during Emergency Department admission–A qualitative inquiry. Australas Emerg Care [Internet]. 2018;21(4):137–42. Available from: doi: 10.1016/j.auec.2018.09.002 30998889

[45] Butragueño Laiseca L, González Martínez F, Oikonomopoulou N, Pérez Moreno J, Toledo del Castillo B, González Sánchez MI, et al. Percepción de los adolescentes sobre el ingreso hospitalario. Importancia de la humanización de los hospitales infantiles. Rev Chil Pediatr. 2016;87(5):373–9. 27181263 10.1016/j.rchipe.2016.04.003

[46] López Naranjo I, Fernández Castillo A. Hospitalización infantil y atención psico-educativa en contextos excepcionales de aprendizaje. Dep Psicol Evol y la Educ Univ Granada [Internet]. 2006;553–77. http://www.ince.mec.es/revistaeducacion/re341/re341_23.pdf

[47] Ullán, Ana M, González-celador R, Manzanera P. El cuidado de los adolescentes en los hospitales. 2010; 10.1016/j.cali.2009.12.006 20207569

[48] Ullán AM, Serrano I, Badía M, Delgado J. Hospitales amigables para adolescentes: Preferencias de los pacientes. Enferm Clin. 2010;20(6):341–8. 20965764 10.1016/j.enfcli.2010.07.006

[49] Generalitat de Catalunya. Departament de Salut. Recomanacions per a la millora de l’atenció de la salut maternoinfantil. Barcelona; 2012.

[50] Lum A, Wakefield CE, Donnan B, Burns MA, Fardell JE, Marshall GM. Understanding the school experiences of children and adolescents with serious chronic illness: a systematic meta-review. Child Care Health Dev. 2017;43(5):645–62. doi: 10.1111/cch.12475 28543609

[51] Shaw SR, McCabe PC. Hospital-to-school transition for children with chronic illness: Meeting the new challenges of an evolving health care system. Psychol Sch [Internet]. 2008 Jan;45(1):74–87. Available from: http://www.ncbi.nlm.nih.gov/pubmed/18708246

[52] Richardson KL, Weiss NS, Halbach S. Chronic School Absenteeism of Children with Chronic Kidney Disease. J Pediatr [Internet]. 2018 Aug;199(1):267–71. Available from: https://linkinghub.elsevier.com/retrieve/pii/S0022347618303780 29706492 10.1016/j.jpeds.2018.03.031 PMC6063782

[53] Emerson ND, Distelberg B, Morrell HER, Williams-Reade J, Tapanes D, Montgomery S. Quality of Life and School Absenteeism in Children With Chronic Illness. J Sch Nurs. 2016;32(4):258–66. doi: 10.1177/1059840515615401 26572160 PMC4867299

[54] Razzaghi H, Oster M, Reefhuis J. Long-Term Outcomes in Children with Congenital Heart Disease: National Health Interview Survey. J Pediatr [Internet]. 2015 Jan;166(1):119–124.e1. Available from: https://linkinghub.elsevier.com/retrieve/pii/S0022347614008208 25304924 10.1016/j.jpeds.2014.09.006 PMC4378575

[55] Riccio CA, Maykel C, Bray MA, Perdue E, Frye S. School Reintegration for Youth with Health-Related Conditions. Contemp Sch Psychol. 2022;26(2):200–8.

[56] Pinquart M. Self-esteem of children and adolescents with chronic illness: A meta-analysis. Child Care Health Dev. 2013;39(2):153–61. doi: 10.1111/j.1365-2214.2012.01397.x 22712715

[57] So SCY, Li WHC, Ho KY. The impact of congenital heart disease on the psychological well-being and quality of life of Hong Kong Chinese adolescents: A cross-sectional study. J Clin Nurs. 2019;28(17–18):3158–67. doi: 10.1111/jocn.14864 30938874

[58] Dahlbeck DT, Lightsey OR. Generalized self-efficacy, coping, and self-esteem as predictors of psychological adjustment among children with disabilities or chronic illnesses. Child Heal Care. 2008;37(4):293–315.

[59] Ferro MA, Boyle MH. The Impact of Chronic Physical Illness, Maternal Depressive Symptoms, Family Functioning, and Self-esteem on Symptoms of Anxiety and Depression in Children. J Abnorm Child Psychol. 2015;43(1):177–87. doi: 10.1007/s10802-014-9893-6 24938212

[60] Ernst MM, Marino BS, Cassedy A, Piazza-Waggoner C, Franklin RC, Brown K, et al. Biopsychosocial Predictors of Quality of Life Outcomes in Pediatric Congenital Heart Disease. Pediatr Cardiol. 2018;39(1):79–88. doi: 10.1007/s00246-017-1730-6 28980091

[61] Pradhan P V., Shah H, Rao P, Ashturkar D, Ghaisas P. Psychopathology and self-esteem in chronic illness. Indian J Pediatr. 2003;70(2):135–8. doi: 10.1007/BF02723739 12661807

[62] Anguera MT, Portell M, Chacón-Moscoso S, Sanduvete-Chaves S. Indirect observation in everyday contexts: Concepts and methodological guidelines within a mixed methods framework. Front Psychol. 2018;9(JAN):1–20. doi: 10.3389/fpsyg.2018.00013 29441028 PMC5797623

[63] Arana J, Lapresa D, Anguera MT, Garzón B. Ad hoc procedure for optimising agreement between observational records. An Psicol. 2016;32(2):589.

[64] Tobin DL, Holroyd KA, Reynolds R V., Wigal JK. The hierarchical factor structure of the coping strategies inventory. Cognit Ther Res. 1989;13(4):343–61.

[65] Creswell JW, Plano Clark VL. Designing and conducting Mixed Methods Research (2nd ed.). Thousand Oaks, CA: Sage.; 2011.

[66] Izquierdo C, Anguera MT. The Analysis of Interpersonal Communication in Sport From Mixed Methods Strategy: The Integration of Qualitative-Quantitative Elements Using Systematic Observation. Front Psychol. 2021;12(March). doi: 10.3389/fpsyg.2021.637304 33868108 PMC8044298

[67] Onwuegbuzie AJ, Teddlie C. A framework for analyzing data in mixed methods research. In: Tashakkori A, Teddlie C, editors. Handbook of Mixed Methods in Social and Behavioral Research. London: SAGE; 2003. p. 351–83.

[68] Ravens-Sieberer U, Bullinger M. Manual KINDL-R. English. Questionnaire for Measuring Health-Related Quality of Life in Children and Adolescents Revised Version. 2000;1–25.

[69] Ravens-Sieberer U, Bullinger M. Cuestionario para niños Kiddy-KINDL-R. 2000;1–3.

[70] Ravens-Sieberer U, Bullinger M. Cuestionario para niños Kid-KINDL-R. 2000;1–4.

[71] Ravens-Sieberer U, Bullinger M. Cuestionario para niños Kiddo-KINDL-R. Suparyanto dan Rosad (2015. 2000;5(3):248–53.

[72] Ravens-Sieberer U, Bullinger M. Cuestionario sobre la calidad de vida de niños y jóvenes, Kiddy-KINDL-R. Versión para los padres. 2000;1–5.

[73] Ravens-Sieberer U, Bullinger M. Cuestionario sobre la calidad de vida de niños y jóvenes, Kid-& Kiddo-KINDL-R. Versión para padres. 2000;1–4.

[74] Fernández-López JA, Fernández Fidalgo M, Cieza A, Ravens-Sieberer U. Medición de la calidad de vida en niños y adolescentes: comprobación preliminar de la validez y fiabilidad de la versión española del cuestionario KINDL. Atención Primaria [Internet]. 2004;33(8):434–42. Available from: 10.1016/S0212-6567(04)79429-9 15151790 PMC7681901

[75] Rajmil L, Serra-Sutton V, Fernandez-Lopez JA, Berra S, Aymerich M, Cieza A, et al. Versión Española del cuestionario Alemán de calidad de vida relacionada con la salud en población infantil y de adolescentes: El Kindl. An Pediatr. 2004;60(6):514–21. 10.1016/s1695-4033(04)78320-4 15207162

[76] Bullinger M, Brütt AL, Erhart M, Ravens-Sieberer U. Psychometric properties of the KINDL-R questionnaire: Results of the BELLA study. Eur Child Adolesc Psychiatry. 2008;17(SUPPL. 1):125–32. doi: 10.1007/s00787-008-1014-z 19132312

[77] Folkman S, Lazarus RS. An Analysis of Coping in a Middle-Aged Community Sample Author (s): Susan Folkman and Richard S. Lazarus Source: Journal of Health and Social Behavior, Vol. 21, No. 3 (Sep., 1980), pp. 219–239 Published by: American Sociological Association S. J Health Soc Behav. 1980;21(3):219–39. 7410799

[78] Rubio L, Dumitrache CG, Cordón-Pozo E, Rubio-Herrera R. Psychometric properties of the Spanish version of the Coping Strategies Inventory (CSI) in elderly people. An Psicol. 2016;32(2):355.

[79] Muñoz-Violant S, Violant-Holz V, Gallego-Jiménez MG, Anguera MT, Rodríguez MJ. Coping strategies patterns to buffer the psychological impact of the State of Emergency in Spain during the COVID-19 pandemic’s early months. Sci Rep [Internet]. 2021;11(1):1–16. Available from: doi: 10.1038/s41598-021-03749-z 34937863 PMC8695586

[80] Guite JW, McCue RL, Sherker JL, Sherry DD, Rose JB. Relationships among pain, protective parental responses, and disability for adolescents with chronic musculoskeletal pain: The mediating role of pain catastrophizing. Clin J Pain. 2011;27(9):775–81. doi: 10.1097/AJP.0b013e31821d8fb4 21593664

[81] Goubert L, Eccleston C, Vervoort T, Jordan A, Crombez G. Parental catastrophizing about their child’s pain. The parent version of the Pain Catastrophizing Scale (PCS-P): A preliminary validation. Pain. 2006;123(3):254–63. doi: 10.1016/j.pain.2006.02.035 16644128

[82] Logan DE, Simons LE, Carpino EA. Too sick for school? Parent influences on school functioning among children with chronic pain. Pain [Internet]. 2012;153(2):437–43. Available from: doi: 10.1016/j.pain.2011.11.004 22169177 PMC3884564

[83] Caes L, Vervoort T, Eccleston C, Vandenhende M, Goubert L. Parental catastrophizing about child’s pain and its relationship with activity restriction: The mediating role of parental distress. Pain [Internet]. 2011;152(1). Available from: https://journals.lww.com/pain/Fulltext/2011/01000/Parental_catastrophizing_about_child_s_pain_and.33.aspx 21126822 10.1016/j.pain.2010.10.037

[84] Kim EJ. Caregiver Stress and Related Factors in Korean Households Utilizing Childcare Support by Grandmothers. Asian Soc Work Policy Rev. 2016;10(1):113–29.

[85] Liu Q, Mo L, Huang X, Yu L, Liu Y. Path analysis of the effects of social support, selfefficacy, and coping style on psychological stress in children with malignant tumor during treatment. Med (United States). 2020;99(43). doi: 10.1097/MD.0000000000022888 33120834 PMC7581179

[86] Mauritz PJ, Bolling M, Duipmans JC, Hagedoorn M. The relationship between quality of life and coping strategies of children with EB and their parents. Orphanet J Rare Dis [Internet]. 2021;16(1):1–9. Available from: doi: 10.1186/s13023-021-01702-x 33516244 PMC7847038

[87] Petersen C, Schmidt S, Bullinger M, Quittan M, Schuhfried O, Nourafza R, et al. Coping with a chronic pediatric health condition and health-related quality of life. Eur Psychol. 2006;11(1):50–6.

[88] Quitmann J, Rohenkohl A, Specht A, Petersen-Ewert C, Schillmöller Z, Bullinger M. Coping Strategies of Children and Adolescents with Clinically Diagnosed Short Stature. J Child Fam Stud. 2015;24(3):703–14.

[89] Schoenmakers EC, van Tilburg TG, Fokkema T. Problem-focused and emotion-focused coping options and loneliness: how are they related? Eur J Ageing. 2015;12(2):153–61. doi: 10.1007/s10433-015-0336-1 28804352 PMC5549139

[90] Willard VW, Hostetter SA, Hutchinson KC, Bonner MJ, Hardy KK. Benefit Finding in Maternal Caregivers of Pediatric Cancer Survivors: A Mixed Methods Approach. J Pediatr Oncol Nurs. 2016;33(5):353–60. doi: 10.1177/1043454215620119 26811326

[91] Grover S, Bhadada S, Kate N, Sarkar S, Bhansali A, Avasthi A, et al. Coping and caregiving experience of parents of children and adolescents with type-1 diabetes: An exploratory study. Perspect Clin Res. 2016;7(1):32. doi: 10.4103/2229-3485.173776 26955574 PMC4763515

[92] Manalel JA, Sumrall S, Davidson H, Grewal M, Granovetter MA, Koehly LM. Stress, coping, and positive aspects of caregiving among caregivers of children with rare disease. Psychol Heal [Internet]. 2022;0(0):1–17. Available from: doi: 10.1080/08870446.2022.2057494 35620936 PMC9701241

[93] Bassi G, Mancinelli E, Di Riso D, Salcuni S. Parental stress, anxiety and depression symptoms associated with self-efficacy in paediatric type 1 diabetes: A literature review. Int J Environ Res Public Health. 2021;18(1):1–20. 10.3390/ijerph18010152 PMC7795592 33379307

[94] Dirks E, Szarkowski A. Family-Centered Early Intervention (FCEI) Involving Fathers and Mothers of Children Who Are Deaf or Hard of Hearing: Parental Involvement and Self-Efficacy. J Clin Med. 2022;11(3). doi: 10.3390/jcm11030492 35159944 PMC8836809

[95] Nap-van der Vlist MM, van der Wal RC, Grosfeld E, van de Putte EM, Dalmeijer GW, Grootenhuis MA, et al. Parent-Child Dyadic Coping and Quality of Life in Chronically Diseased Children. Front Psychol. 2021;12(July):1–8. doi: 10.3389/fpsyg.2021.701540 34393938 PMC8355494

[96] Birkeland B, Weimand B, Ruud T, Maybery D, Vederhus JK. Perceived family cohesion, social support, and quality of life in patients undergoing treatment for substance use disorders compared with patients with mental and physical disorders. Addict Sci Clin Pract [Internet]. 2021;16(1):1–9. Available from: doi: 10.1186/s13722-021-00252-8 34193283 PMC8246687

[97] Bradshaw S, Bem D, Shaw K, Taylor B, Chiswell C, Salama M, et al. Improving health, wellbeing and parenting skills in parents of children with special health care needs and medical complexity—A scoping review. BMC Pediatr. 2019;19(1):1–11. 31470820 10.1186/s12887-019-1648-7 PMC6716943

[98] Bodenmann G. Dyadic coping and the significance of this concept for prevention and therapy. Zeitschrift für Gesundheitspsychologie [Internet]. 2008 Jul;16(3):108–11. Available from: https://econtent.hogrefe.com/doi/10.1026/0943-8149.16.3.108

[99] Schmied V, Mills A, Kruske S, Kemp L, Fowler C, Homer C. The nature and impact of collaboration and integrated service delivery for pregnant women, children and families. J Clin Nurs. 2010;19(23–24):3516–26. doi: 10.1111/j.1365-2702.2010.03321.x 20946442

[100] Hill C, Knafl KA, Santacroce SJ. Family-Centered Care from the Perspective of Parents of Children Cared for in a PICU: An Integrative Review. Physiol Behav. 2019;176(1):139–48. 10.1016/j.pedn.2017.11.007 PMC5955783 29153934

[101] Khan A, Spector ND, Baird JD, Ashland M, Starmer AJ, Rosenbluth G, et al. Patient safety after implementation of a coproduced family centered communication programme: Multicenter before and after intervention study. BMJ. 2018;363. doi: 10.1136/bmj.k4764 30518517 PMC6278585

[102] Hauskov Graungaard A, Hafting M, Davidsen AS, Lykke K. How is my child doing–parental understanding of their children when a parent has cancer. J Psychosoc Oncol [Internet]. 2021;0(0):1–16. Available from: doi: 10.1080/07347332.2021.2013386 34961424

[103] Cena L, Biban P, Janos J, Lavelli M, Langfus J, Tsai A, et al. The Collateral Impact of COVID-19 Emergency on Neonatal Intensive Care Units and Family-Centered Care: Challenges and Opportunities. Front Psychol. 2021;12(February):1–10. doi: 10.3389/fpsyg.2021.630594 33716895 PMC7943863

[104] Sawyer JL, Mishna F, Bouffet E, Saini M, Zlotnik-Shaul R. Bridging the Gap: Exploring the Impact of Hospital Isolation on Peer Relationships Among Children and Adolescents with a Malignant Brain Tumor. Child Adolesc Soc Work J [Internet]. 2021;(0123456789). Available from: doi: 10.1007/s10560-021-00764-x 34025015 PMC8130807

[105] Violant-Holz V, Salmeron C, Ponce C. Quality of life in childhood with congenital heart disease. New Educ Rev. 2012;27(1):64–77.

[106] Tenniglo LJA, Loeffen EAH, Kremer LCM, Font-Gonzalez A, Mulder RL, Postma A, et al. Patients’ and parents’ views regarding supportive care in childhood cancer. Support Care Cancer. 2017;25(10):3151–60. doi: 10.1007/s00520-017-3723-7 28456909 PMC5577054

[107] Leary JC, Krcmar R, Yoon GH, Freund KM, LeClair AM. Parent Perspectives During Hospital Readmissions for Children With Medical Complexity: A Qualitative Study. Hosp Pediatr. 2020;10(3):222–9. doi: 10.1542/hpeds.2019-0185 32029432 PMC7041550

[108] Gómez-Gamboa E, Rodrigo-Pedrosa O, San-Millán M, Saz-Roy MA, Negre-Loscertales A, Puig-Llobet M. The Perceptions of Children and Adolescents with Cancer Regarding Nurses’ Communication Behaviors during Needle Procedures. Int J Environ Res Public Health. 2022;19(15):1–12. doi: 10.3390/ijerph19159372 35954729 PMC9368135

[109] Carosella A, Snyder A, Ward E. What parents of children with complex medical conditions want their child’s physicians to understand. JAMA Pediatr. 2018;172(4):315–6. doi: 10.1001/jamapediatrics.2017.3931 29404579

